# Association of front-of-package warning label perceptions with Mediterranean diet adherence after bariatric surgery: FOP perceptions and MedDiet adherence post-bariatric surgery

**DOI:** 10.1016/j.obpill.2026.100266

**Published:** 2026-04-14

**Authors:** Sila Maribel Laboriano Milián, Lesly Pamela Palomino Ruiz, Wilter C. Morales-García, Yaquelin E. Calizaya-Milla

**Affiliations:** a Unidad de Salud Pública, Escuela de Posgrado, Universidad Peruana Unión, Lima, Peru; b Dirección General de Investigación, Universidad Peruana Unión, Lima, Peru; c Research Group for Nutrition and Lifestyle, Escuela de Nutrición Humana, Facultad de ciencias de la Salud, Universidad Peruana Unión, Lima, 15, Peru

**Keywords:** Bariatric surgery, Front-of-package warning labels, Mediterranean diet, Obesity, Dietary adherence

## Abstract

**Background:**

Front-of-package (FOP) warning labels are intended to support healthier food choices; however, evidence on how patients perceive these warnings after bariatric surgery and how such perceptions relate to dietary quality is limited. We examined the association between perceptions of FOP warning labels and Mediterranean diet (MedDiet) adherence among adults after bariatric surgery in Lima, Peru.

**Methods:**

This was an observational, analytical, cross-sectional study of 200 adults (18–59 years) attending postoperative follow-up at a private clinic in Lima, Peru. MedDiet adherence was assessed using the KIDMED index (originally developed for children/adolescents and used here as a proxy measure of MedDiet-aligned behaviors), and perceptions/understanding of FOP octagonal warnings were measured using a previously validated Peruvian questionnaire. Associations were evaluated using bivariate analyses and multivariable logistic regression, reporting adjusted odds ratios (aOR) and 95% confidence intervals (CI).

**Results:**

In adjusted models, higher self-reported preference for salty foods (aOR = 0.261; 95% CI: 0.136–0.501) and sweet foods (aOR = 0.490; 95% CI: 0.263–0.913) were associated with lower odds of medium/high MedDiet adherence. Greater agreement with the statement “I would follow the octagons, but nobody guides me on how to use them” was associated with higher odds of medium/high MedDiet adherence (aOR = 1.289; 95% CI: 1.002–1.659).

**Conclusion:**

Among adults after bariatric surgery, taste-preference indicators and perceptions regarding FOP warning labels were independently associated with MedDiet adherence. Given the cross-sectional design, these findings should be interpreted as associations rather than causal relationships and highlight the need to strengthen label literacy during postoperative nutrition education.

## Graphical abstract

**Figure:**
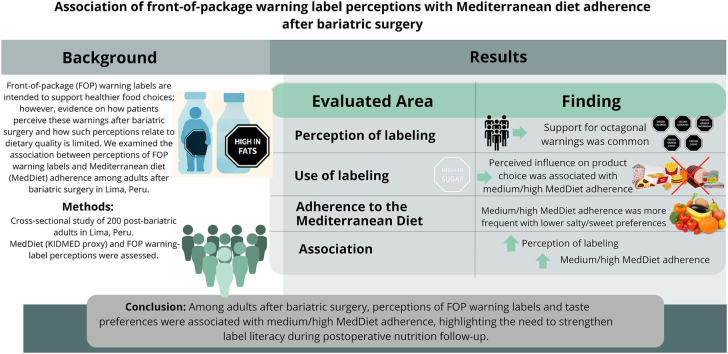

## 1 Introduction

Obesity is a global health priority because of its high prevalence and the clustering of comorbidities—including type 2 diabetes, hypertension, obstructive sleep apnea, and several cancers—that collectively impair functioning, diminish quality of life, and increase premature mortality [1]. In Latin America, the prevalence of excess body weight has risen steadily and now affects most adults, within a food environment characterized by high availability of ultra-processed products and low consumption of fruits and vegetables [2,3]. The national picture mirrors this trend: in Peru, 6 in 10 individuals aged ≥15 years have excess body weight, and fewer than 1 in 10 meet the recommendation of five daily servings of fruits and/or vegetables, with a disproportionate burden among women [4].

Against this backdrop, bariatric surgery has emerged as the most effective intervention for severe obesity, reducing all-cause mortality and major cardiometabolic events, with evidence from long-term cohort studies such as the Swedish Obese Subjects (SOS) study [5,6]. Nevertheless, medium- and long-term success depends critically on the quality of the dietary pattern patients can sustain in the postoperative period, during which substantial metabolic benefits coexist with a non-trivial vulnerability to micronutrient and protein deficiencies due to restriction, malabsorption, and changes in food tolerance [7,8]. This dual reality—cardiometabolic improvement alongside nutritional risk—underscores the need to identify dietary frameworks that are both cardioprotective and adoptable and sustainable after surgery [7].

The Mediterranean Diet (MedDiet) meets these criteria given its extensive clinical and epidemiological support. Randomized trials such as PREDIMED have demonstrated reductions in major cardiovascular events in primary prevention, while large-scale prospective studies and meta-analyses link higher adherence to lower all-cause mortality and consistent improvements in systemic inflammation, insulin resistance, and an atherogenic lipid profile [9,10]. In the post-bariatric setting, its architecture—high intake of fruits, vegetables, legumes, nuts, and olive oil; a preference for monounsaturated fats; and restriction of added sugars and trans fats—aligns with goals of recovery, preservation of lean mass, and prevention of deficiencies, and it facilitates texture, volume, and meal-fractionation adjustments typical of the postoperative period [10]. Emerging evidence further suggests that greater MedDiet adherence among patients undergoing bariatric surgery is associated with greater one-year weight loss and better overall diet quality, reinforcing its role as a nutritional scaffold to consolidate outcomes [11,12].

In Peru, front-of-package labeling is implemented through black octagonal warning labels placed on packaged foods and beverages that exceed thresholds for sugar, sodium, saturated fat, or contain trans fats [13,14]. These warnings are intended to provide a rapid interpretive cue at the point of purchase. Translating the intention to “eat better” into coherent daily behaviors, however, requires navigating rapid decisions in an environment saturated with commercial cues and low-nutrient-density products. In this context, front-of-package (FOP) nutrition labeling systems are especially relevant because they provide a simple, visible, and interpretive cue at the point of decision, without requiring advanced nutrition literacy [15,16]. Regulatory experience supports their utility. In Chile, the phased implementation of octagonal “HIGH IN” warning labels was associated with measurable reductions in purchases of beverages and foods high in critical nutrients, suggesting that warning labels reshape demand at the population level [17,18]. In Europe, experimental and quasi-experimental evaluations of Nutri-Score show advantages in objective understanding and the nutritional quality of the “shopping basket” compared with numeric schemes or multiple-traffic-light systems, including among individuals with cardiometabolic conditions [15,16]. Likewise, recent comparative analyses indicate that when the goal is to discourage high-risk nutritional products, warning systems tend to outperform global summaries or traffic-light formats—an especially relevant consideration for secondary prevention [19].

Among post-bariatric patients, front-of-pack (FOP) nutrition labeling may function as a choice heuristic that narrows the gap between clinical recommendations and real-world purchasing behavior. Three arguments support this hypothesis. First, the postoperative period involves sensory and behavioral relearning—small portions, tolerated textures, and aversion to simple sugars and fats—that renders routines fragile; clear front-of-pack cues can buffer lapses and impulses, reinforcing the selection of higher–nutrient-density options [7]. Second, given the risk of protein and micronutrient deficiencies, an effective FOP system could encourage the choice of minimally processed, higher-quality foods aligned with the pillars of the MedDiet [7,20]. Third, in a high-intensity marketing environment, embedding validated signals into the shopping experience may counter persuasive advertising and support self-regulation, particularly during phases of fluctuating appetite and satiety [21].

That said, the mere presence of FOP labeling does not ensure uniform uptake or equivalent effects across groups. The literature documents variability in comprehension, trust, and use, as well as debate about the magnitude of real-world effects and the sensitivity of labeling algorithms to reformulation and specific product categories [22,23]. This heterogeneity may be even greater among bariatric patients, who follow supplementation regimens, periodic check-ups, and individualized recommendations: some may view FOP labeling as a complementary tool that simplifies decisions, whereas others may consider it redundant or not credible, with direct implications for adherence to healthy patterns [24]. Accordingly, perception of FOP labeling—understood as its perceived credibility, clarity, and usefulness—may act as a critical moderator between nutrition education and actual purchasing behavior aligned with the MedDiet [16,17].

The regional regulatory landscape also offers a natural experiment. Across Latin America, several countries—including Ecuador, Chile, and Peru—have implemented front-of-pack (FOP) labeling schemes over the past decade, creating settings in which it is possible to assess not only comprehension but also the real-world adoption of healthier food choices within specific clinical groups [25]. In Peru, FOP labeling is embedded in the national policy to promote healthy eating; however, consumer surveys indicate that label reading and understanding remain limited and heterogeneous, with notable levels of distrust [26]. This mismatch between signal availability and effective uptake reinforces the need to examine perceptions of FOP labeling among patients who, by virtue of their condition, stand to benefit especially from high–nutrient-quality choices.

Finally, although evidence on front-of-pack (FOP) labeling is encouraging at the population level, important gaps remain for bariatric patients. In addition, FOP systems differ across countries and are periodically updated, altering product classifications and, potentially, consumer decisions [15,27]. Context-specific studies that integrate BMI status, perception of FOP labeling, and adherence to the MedDiet in the postoperative setting can generate evidence directly applicable to clinical practice and public policy, thereby optimizing the continuum of care after surgery [18].

Although many studies have assessed adults’ BMI status, few have evaluated how front-of-pack (FOP) nutrition labeling is used and how its use relates to diet quality in this population. Accordingly, this study aimed to examine the association between perceptions of front-of-package warning labels and MedDiet adherence among adults after bariatric surgery.

## 2 Methods

### 2.1 Study design and participants

We conducted an observational, analytical, cross-sectional study using an online questionnaire and an in-person interview administered to postoperative bariatric patients at a private clinic in Pueblo Libre, Lima, Peru. The surgical procedures represented in the sample were sleeve gastrectomy and Roux-en-Y gastric bypass. The questionnaires were administered one month after the surgical intervention, during the postoperative nutritional consultation. The protocol was approved by the clinic's Research Ethics Committee prior to data collection (2025-CE-EPG-00046), in accordance with the Declaration of Helsinki and local regulations. Participant confidentiality was maintained throughout the study. A non-probability convenience sampling approach was used. The minimum sample size was estimated a priori with Soper's online calculator (version 4.0, 2024) for multiple-predictor models, assuming a medium anticipated effect size (f^2^ = 0.15), α = 0.05, statistical power = 0.80, and 14 predictors, yielding a required n = 135. To increase precision and accommodate potential exclusions/nonresponse, we targeted and enrolled 200 bariatric patients aged ≥18 years.

### 2.2 Variables and data sources

Sociodemographic, anthropometric, and surgical data were collected using an ad hoc standardized case report form. The form captured age, sex, educational level, marital status, weight, height, type of bariatric procedure, and operative information when available.

### 2.3 BMI status

Body mass index (BMI) was calculated as weight (kg)/height (m^2^) and categorized according to WHO adult cutoffs as underweight (<18.5 kg/m^2^), normal weight (18.5–24.9 kg/m^2^), overweight (25.0–29.9 kg/m^2^), and obesity (≥30.0 kg/m^2^). In this study, BMI was used as an anthropometric indicator of weight status rather than as a comprehensive measure of nutritional status.

### 2.4 Perceived excess weight

Perceived excess weight was assessed through a single self-reported item that inquired whether participants considered themselves to have excess body weight. The responses were coded dichotomously, with “no” assigned a value of 0 and “yes” assigned a value of 1. This variable reflects the participant's subjective perception of their own weight status, which may not necessarily correspond to objective anthropometric measurements but provides relevant insight into body image and self-perception related to weight [28]. Saltiness and sweetness preference were assessed as self-reported taste preference categories, classified as little/not salty versus very/regularly salty, and little/not sweet versus very/regularly sweet.

### 2.5 Diet quality (MedDiet adherence)

Diet quality was assessed using the KIDMED index, a 16-item questionnaire with dichotomous response options (yes/no) [29]. Affirmative responses to the 12 items reflecting Mediterranean-diet-aligned behaviors (e.g., fruit, vegetables, fish, legumes, cereals, nuts, olive oil, and dairy products) were scored +1, whereas affirmative responses to the 4 items reflecting less healthy eating behaviors (e.g., fast food, skipping breakfast, pastries/cookies, and sweets/candy) were scored −1. Negative responses were scored 0. The total score, obtained by summing all 16 items, ranges from −4 to 12.

For the purposes of this study, KIDMED scores were dichotomized as low adherence (≤3 points) and medium/high adherence (4–12 points), so that the analytical outcome reflected less favorable versus more favorable Mediterranean-diet-related behaviors. Because the KIDMED index was originally developed for children and adolescents, it was used here as a proxy measure of Mediterranean-diet-aligned behaviors in adults after bariatric surgery. In addition, a preliminary internal psychometric evaluation was conducted in the present sample. The KIDMED index showed a KR-20 of 0.55 and an ordinal alpha of 0.67, indicating modest internal consistency. Therefore, findings derived from this instrument should be interpreted with caution.

### 2.6 Front-of-Pack (FOP) labeling perception

Perception of FOP labeling, purchase tendency, and ultra-processed food consumption were assessed using a questionnaire developed and validated in Peru [30]. The instrument comprises ten closed-ended items on a five-point Likert scale (strongly disagree, disagree, neither agree nor disagree, agree, strongly agree) capturing clarity, credibility, and perceived usefulness of FOP labels and their influence on purchase intention. It also includes five additional items covering preference for sweet/salty taste and presence of noncommunicable diseases (NCDs) (hypertension, diabetes mellitus, dyslipidemias—cholesterol, triglycerides, or both). For analysis, NCD presence was coded no = 0, yes = 1. The instrument showed Cronbach's alpha = 0.69, indicating moderate internal consistency, and has been reported as culturally appropriate for Peruvian populations.

The KIDMED item structure and the front-of-package warning-label perception questionnaire used in this study are provided in Supplementary Tables S1 and S2.

### 2.7 Statistical analysis

Analyses were performed in IBM SPSS Statistics v28. Continuous variables were summarized as mean ± SD or median [IQR] according to distribution (assessed with Shapiro–Wilk and graphical diagnostics); categorical variables as counts and percentages. Bivariate associations between MedDiet adherence and covariates were examined using χ^2^ or Fisher's exact tests for categorical variables and the Mann–Whitney *U* test for non-normally distributed continuous variables; Spearman's ρ was used when appropriate. A binary logistic regression model was fitted with medium/high adherence as the outcome (KIDMED 4–12 = 1 vs ≤ 3 = 0). Candidate predictors included sociodemographic, clinical, and FOP-label perception variables. We applied stepwise backward (Wald) selection to screen predictors and then refit a parsimonious forced-entry (Enter) model including the significant variables. Multicollinearity was assessed (VIF<5); model calibration and discrimination were evaluated using the Hosmer–Lemeshow test and area under the ROC curve (AUC). Results are reported as odds ratios (ORs) with 95% confidence intervals (CIs). All tests were two-sided with α = 0.05. Missing data were handled via complete-case (listwise) analysis.

As a preliminary internal psychometric assessment of the KIDMED index in this sample, the 16 items were recoded into a common healthy orientation, with reverse coding applied to negatively worded items. Internal consistency was examined using the Kuder-Richardson Formula 20 (KR-20) and ordinal alpha based on tetrachoric correlations. Corrected item-total correlations and exploratory dimensionality indices were also inspected.

## 3 Results

Table 1 summarizes baseline characteristics. Participants had a mean age of 36.9 years, and 62.5% were women. University education was reported by 41.5%, and 65.5% were single. The sample included adults who had undergone sleeve gastrectomy or Roux-en-Y gastric bypass, and the questionnaires were administered one month after surgery during the postoperative nutritional consultation. Regarding taste preferences, 61.0% reported eating foods that were little or not salty, and 62.5% preferred foods that were little or not sweet. Overall, 65.0% had excess body weight. Dyslipidemia, hypertension, and type 2 diabetes were present in 39.5%, 12.0%, and 8.5% of participants, respectively.

**Table 1:** Sociodemographic and clinical characteristics (n = 200).

Variable	Category	n (%),Mean ± SD
Age (years)		36,9 ± 11.54
Sex	Female	125 (62.5)
	Male	75 (37.5)
Education	Primary/Basic	49 (24.5)
	Technical/Vocational	68 (34.0)
	University	83 (41.5)
Marital status	Single	131 (65.5)
	Married	63 (31.5)
	Divorced	6 (3.0)
Saltiness preference	Little/not salty	122 (61.0)
	Very/regularly salty	78 (39.0)
Sweetness preference	Little/not sweet	125 (62.5)
	Very/regularly sweet	75 (37.5)
Hypertension	Yes	24 (12.0)
	No	176 (88.0)
Type 2 diabetes	Yes	17 (8.5)
	No	183 (91.5)
Dyslipidemia	Yes	79 (39.5)
	No	121 (60.5)
BMI	Normal weight	70 (35.0)
	Overweight	99 (49.5)
	Obesity	31 (15.5)

SD = Standard Deviation; BMI = Body Mass Index.

As a preliminary internal psychometric assessment in this sample, the KIDMED index showed a KR-20 of 0.55 and an ordinal alpha of 0.67, indicating modest internal consistency. Bartlett's test of sphericity was significant (χ^2^[120] = 446.83, p < 0.001), and the Kaiser-Meyer-Olkin index was 0.57, suggesting limited but acceptable factorability for exploratory purposes. However, the internal structure did not support a clearly unidimensional solution, so the KIDMED results should be interpreted as a proxy indicator of Mediterranean-diet-aligned behaviors rather than as evidence of full validation in adults after bariatric surgery.

Regarding perceptions of front-of-package warning labels, 44.0% of participants agreed that the octagonal warning label influenced their product choice, and 38.5% reported that prices and promotions also affected purchase decisions. In terms of perceived barriers, 30.5% indicated that they did not know how to interpret the octagons, and 31.5% stated that they received no guidance on how to use them. Support for the policy was also notable: 34.0% strongly agreed with the implementation of octagons in Peru, and 34.5% agreed with the label design. Knowledge of recommended intake levels was limited, with only 24.0% reporting knowledge of salt recommendations, 25.5% of sugar recommendations, and 25.0% of saturated fat recommendations (Table 2).

**Table 2:** Perceptions of front-of-package warning labels and nutrition knowledge (n = 200).

Item/question	Strongly disagree	Disagree	Neither agree nor disagree	Agree	Strongly agree
The octagonal warning label on the package influences my product choice	17 (8.5)	29 (14.5)	47 (23.5)	88 (44)	19 (9.5)
Prices and promotions influence my purchase decisions	20 (10)	33 (16.5)	43 (21.5)	77 (38.5)	27 (13.5)
I would follow the octagons, but I do not have time to read them	18 (9)	48 (24)	57 (28.5)	59 (29.5)	18 (9)
I would follow the octagons, but I do not know how to interpret them	26 (13)	47 (23.5)	43 (21.5)	61 (30.5)	23 (11.5)
I would follow the octagons, but I receive no guidance on how to use them	19 (9.5)	44 (22)	45 (22.5)	63 (31.5)	29 (14.5)
I agree with the implementation of octagons in Peru	3 (1.5)	28 (14)	36 (18)	65 (32.5)	68 (34)
I am satisfied with the label design/model	11 (5.5)	35 (17.5)	44 (22)	69 (34.5)	41 (20.5)
I know the recommended intake level for salt	22 (11)	38 (19)	69 (34.5)	48 (24)	23 (11.5)
I know the recommended intake level for sugar	17 (8.5)	58 (29)	54 (27)	51 (25.5)	20 (10)
I know the recommended intake level for saturated fat	17 (8.5)	50 (25)	58 (29)	50 (25)	25 (12.5)

Note: Values are presented as n (%).

In bivariate chi-square analyses, saltiness preference was significantly associated with MedDiet adherence (p < 0.01): 72.3% of participants reporting little/not salty foods showed medium/high adherence, compared with 27.7% among those reporting very/regularly salty foods. Sweetness preference showed a similar association (p = 0.010): 69.7% of participants in the little/not sweet group versus 30.3% in the very/regularly sweet group showed medium/high adherence. For BMI status, medium/high adherence was more frequent among participants with normal weight (42.0%) than among those with obesity (8.4%) (p < 0.01). No significant differences in MedDiet adherence were observed according to sex, educational level, marital status, hypertension, type 2 diabetes, or dyslipidemia (all p > 0.05) (Table 3).

**Table 3:** MedDiet adherence by sociodemographic and clinical variables (n = 200).

MEDDIET ADHERENCE				
Variables	Total	Low	Medium/High	*p-value*
**Sex**				
Female	125 (62.5)	47 (58)	78 (65.5)	0.281
Male	75 (37.5)	34 (42)	41 (34.5)	
**Education**				
Primary/basic	49 (24.5)	20 (24.7)	29 (24.4)	0.983
Technical/vocational	68 (34)	28 (34.6)	40 (33.6)	
University	83 (41.5)	33 (40.7)	50 (42)	
**Marital status**				
Single	131 (65.5)	50 (61.7)	81 (68.1)	0.542
Married	63 (31.5)	29 (35.8)	34 (28.6)	
Divorced	6 (3)	2 (2.5)	4 (3.4)	
**Saltiness preference**				
Little/not salty	122 (61)	36 (44.4)	86 (72.3)	<0.01^b^
Very/regularly salty	78 (39)	45 (55.6)	33 (27.7)	
**Sweetness preference**				
Little/not sweet	125 (62.5)	42 (51.9)	83 (69.7)	0.010^a^
Very/regularly sweet	75 (37.5)	39 (48.1)	36 (30.3)	
**Hypertension**				
Yes	24 (12)	10 (12.3)	14 (11.8)	0.901
No	176 (88)	71 (87.7)	105 (88.2)	
**Type 2 diabetes**				
Yes	17 (8.5)	6 (7.4)	11 (9.2)	0.648
No	183 (91.5)	75 (92.6)	108 (90.8)	
**Dyslipidemia**				
Yes	79 (39.5)	36 (44.4)	43 (36.1)	0.238
No	121 (60.5)	45 (55.6)	76 (63.9)	
**Body mass index**				
Normal weight	70 (35)	20 (24.7)	50 (42)	<0.01 ^b^
Overweight	99 (49.5)	40 (49.4)	59 (49.6)	
Obesity	31 (15.5)	21 (25.9)	10 (8.4)	

Notes: p-values were obtained using chi-square tests (or Fisher's exact test, as appropriate). Adherence was dichotomized as low (KIDMED ≤3) and medium/high (KIDMED 4–12). *p < 0.05; **p < 0.01.

Significant differences were observed according to sex and perceived excess weight across several individual KIDMED items (Table 4). Compared with men, women more often reported legume consumption more than once per week (p < 0.01) and pasta or rice intake almost daily (p < 0.01), but also a higher frequency of pastries/cookies at breakfast (p = 0.046) and a lower frequency of daily yogurt/cheese intake (p = 0.043). Participants who did not perceive themselves as having excess weight more frequently reported breakfast cereals or grains (p = 0.027), legumes more than once per week (p = 0.012), pasta or rice almost daily (p = 0.026), and a dairy product at breakfast (p = 0.035) than those who did perceive excess weight. No significant differences were detected according to the presence of noncommunicable diseases (all p > 0.05). The full wording of the KIDMED items and the FOP warning-label perception items is available in the Supplementary Material.

In this reduced model, higher saltiness preference was inversely associated with medium/high adherence (OR = 0.261; 95% CI: 0.136–0.501; p < 00.001), as was higher sweetness preference (OR = 0.490; 95% CI: 0.263–0.913; p = 00.025). Hypertension showed a positive but non-significant association (p = 00.078). Agreement with the statement “I would follow the octagons, but nobody guides me on how to use them” was significantly associated with higher odds of medium/high adherence (OR = 1.289; 95% CI: 1.002–1.659; p = 00.048). The model was statistically significant overall (χ^2^(4) = 27.351, p < 00.001), with Nagelkerke R^2^ = 0.173 and overall accuracy = 63.0%. Collectively, dietary taste patterns (saltiness/sweetness) and FOP-label perceptions are independently related to MedDiet adherence in this sample (Table 7).

**Table 4:** Comparison of individual KIDMED items according to sex, perceived excess weight, and presence of noncommunicable diseases.

KIDMED item	Men (n = 75)		Women (n = 125)		U statistic	p	Perceived EW (n = 130)		No perceived EW (n = 70)		U statistic	p	NCD present (n = 86)		NCD absent (n = 114)		U statistic	p
	Mean	SD	Mean	SD			Mean	SD	Mean	SD			Mean	SD	Mean	SD		
Fruit or fruit juice every day	0.744	0.438	0.627	0.487	4137.5	0.080	0.669	0.472	0.757	0.432	4950	0.197	0.698	0.462	0.702	0.460	4922	0.950
A second fruit every day	0.520	0.502	0.467	0.502	4437.5	0.466	0.492	0.502	0.514	0.503	4650	0.767	0.430	0.498	0.553	0.499	5502	0.087
Fresh or cooked vegetables once a day	0.672	0.471	0.640	0.483	4537.5	0.645	0.631	0.484	0.714	0.455	4930	0.236	0.640	0.483	0.675	0.470	5078	0.597
Fresh or cooked vegetables more than once a day	0.328	0.471	0.440	0.500	5212.5	0.113	0.354	0.480	0.400	0.493	4760	0.520	0.337	0.476	0.395	0.491	5184	0.405
Fish regularly, at least 2–3 times/week	0.448	0.499	0.387	0.490	4400.0	0.397	0.454	0.500	0.371	0.487	4175	0.262	0.500	0.503	0.368	0.485	4257	0.063
Fast-food restaurant more than once/week	−0.504	0.502	−0.640	0.483	4050.0	0.062	−0.562	0.498	−0.543	0.502	4635	0.800	−0.558	0.500	−0.553	0.499	4929	0.938
Legumes more than once/week	0.488	0.502	0.693	0.464	5650.0	<0.01**	0.500	0.502	0.686	0.468	5395	0.012*	0.547	0.501	0.579	0.496	5061	0.648
Pasta or rice almost daily	0.488	0.502	0.693	0.464	5650.0	<0.01**	0.508	0.502	0.671	0.473	5295	0.026*	0.523	0.502	0.596	0.493	5261	0.302
Cereals or grains for breakfast	0.616	0.488	0.520	0.503	4237.5	0.184	0.523	0.501	0.686	0.468	5290	0.027*	0.593	0.494	0.570	0.497	4790	0.746
Nuts regularly, at least 2–3 times/week	0.384	0.488	0.360	0.483	4575.0	0.735	0.369	0.484	0.386	0.490	4625	0.819	0.384	0.489	0.368	0.485	4827	0.825
Olive oil used at home	0.360	0.482	0.347	0.479	4625.0	0.849	0.362	0.482	0.343	0.478	4465	0.793	0.326	0.471	0.377	0.487	5155	0.451
Skips breakfast	−0.448	0.499	−0.533	0.502	4287.5	0.243	−0.523	0.501	−0.400	0.493	5110	0.097	−0.547	0.501	−0.430	0.497	5474	0.103
Dairy product for breakfast	0.656	0.477	0.547	0.501	4175.0	0.125	0.562	0.498	0.714	0.455	5245	0.035*	0.651	0.479	0.588	0.494	4591	0.363
Pastries/cookies for breakfast	−0.288	0.455	−0.427	0.498	4037.5	0.046*	−0.369	0.484	−0.286	0.455	4930	0.236	−0.407	0.494	−0.289	0.456	5478	0.083
Two yogurts and/or some cheese daily	0.480	0.502	0.333	0.475	4000.0	0.043*	0.423	0.496	0.429	0.498	4575	0.940	0.488	0.503	0.377	0.487	4357	0.116
Sweets/candy several times per day	−0.304	0.462	−0.427	0.498	4112.5	0.079	−0.362	0.482	−0.329	0.473	4700	0.642	−0.360	0.483	−0.342	0.477	4992	0.788

SD = Standard Deviation; EW = Excess Weight; NCD = Noncommunicable Diseases; *p < 0.05; **p < 0.01.

Significant between-group differences were observed across several front-of-package warning-label perception items (Table 5). Participants who did not perceive themselves as having excess weight scored higher on the item indicating that the octagonal warning label influences product choice (p < 0.01). Regarding noncommunicable disease status, participants without noncommunicable diseases scored higher on the items related to the influence of the octagonal warning label on product choice (p < 0.01), the influence of prices and promotions on purchase decisions (p < 0.01), agreement with the implementation of octagons in Peru (p < 0.01), and satisfaction with the label design/model (p < 0.01). In addition, women scored higher than men on satisfaction with the label design/model (p = 0.015). No significant differences were observed for the remaining front-of-package warning-label perception items (all p > 0.05). Higher scores indicate stronger agreement on the 5-point Likert scale.

**Table 5:** Independent-samples comparison of means—Student's *t*-test (Mann–Whitney U)—for front-of-pack (FOP) nutrition labeling perception by sex, perceived excess weight, and presence of NCDs.

FOP item	Men (n = 75)		Women (n = 125)		U statistic	p	Perceived EW (n = 130)		No perceived EW (n = 70)		U statistic	p	NCD present (n = 86)		NCD absent (n = 114)		U statistic	P
	Mean	SD	Mean	SD			Mean	SD	Mean	SD			Mean	SD	Mean	SD		
The octagonal warning label on the package influences my product choice	3.280	1.126	3.373	1.063	4843.0	0.679	3.162	1.119	3.600	1.013	5597.0	<0.01**	2.942	1.099	3.596	1.019	6472.5	<0.01**
Prices and promotions influence my purchase decisions	3.176	1.245	3.480	1.070	5239.0	0.148	3.192	1.233	3.471	1.086	5096.5	0.146	2.965	1.260	3.535	1.074	6124.5	<0.01**
I would follow the octagons, but I do not have time to read them	2.936	1.113	3.253	1.116	5425.0	0.054	3.031	1.120	3.100	1.131	4641.5	0.809	3.093	1.081	3.026	1.156	4735.0	0.670
I would follow the octagons, but I do not know how to interpret them	2.960	1.221	3.173	1.256	5247.0	0.233	3.131	1.210	2.871	1.273	4041.5	0.180	3.047	1.105	3.035	1.330	4908.5	0.987
I would follow the octagons, but nobody guides me on how to use them	3.192	1.209	3.200	1.219	4609.5	0.839	3.123	1.175	3.329	1.271	5009.0	0.226	3.151	1.173	3.228	1.241	5069.5	0.670
I agree with the implementation of octagons in Peru	3.744	1.106	3.987	1.059	5281.0	0.118	3.662	1.124	4.157	0.958	5696.5	<0.01**	3.453	1.081	4.123	1.014	6641.0	<0.01**
I am satisfied with the label design/model	3.312	1.174	3.733	1.095	5616.0	0.015*	3.323	1.156	3.743	1.125	5487.0	0.013*	2.988	1.222	3.833	0.968	6812.5	<0.01**
I know the recommended intake level for salt	3.080	1.161	3.027	1.150	4667.0	0.958	3.077	1.125	3.029	1.215	4511.5	0.919	3.000	1.158	3.105	1.155	5092.0	0.628
I know the recommended intake level for sugar	3.032	1.135	2.933	1.143	4513.0	0.650	3.023	1.191	2.943	1.034	4457.0	0.806	2.919	1.150	3.053	1.128	5226.0	0.409
I know the recommended intake level for saturated fat	3.128	1.143	3.000	1.185	4460.5	0.555	3.100	1.167	3.043	1.148	4443.5	0.779	3.116	1.100	3.053	1.204	4698.5	0.605

SD = Standard Deviation; EW = Excess Weight; NCD = Noncommunicable Diseases; *p < 0.05; **p < 0.01.

Examining the association between MedDiet adherence (KIDMED: low vs medium/high) and front-of-pack (FOP) label perception, only one item reached statistical significance (p < 0.05): “the octagonal warning on the package influences my product choice.” Participants with medium/high adherence showed a higher proportion of agreement with this statement compared with those with low adherence. All other items were non-significant (all p > 0.05) (Table 6).

**Table 6:** MedDiet adherence and front-of-pack labeling perception (item: “the octagon influences my product choice”).

MedDiet adherence				
FOP label perception	Total n (%)	Low adherence n (%)	Medium/high adherence n (%)	p
Strongly disagree	17 (8.5%)	7 (8.6%)	10 (8.4%)	0.04*
Disagree	29 (14.5%)	18 (22.2%)	11 (9.2%)	
Neither agree nor disagree	47 (23.5%)	12 (14.8%)	35 (29.4%)	
Agree	88 (44%)	36 (44.4%)	52 (43.7%)	
Strongly agree	19 (9.5%)	8 (9.9%)	11 (9.2%)	

Notes: p-values were obtained using the chi-square test. Adherence was dichotomized as low (KIDMED ≤3) and medium/high (KIDMED 4–12). *p < 0.05; **p < 0.01.

Because determinants were not known a priori, we fitted a binary logistic regression using backward (Wald) selection with 20 candidate predictors (sociodemographic and FOP-label perception variables). Based on the final step, we refit a parsimonious forced-entry model including four predictors: saltiness level, sweetness level, hypertension, and agreement with “I would follow the octagons, but nobody guides me on how to use them”.

**Table 7:** Binary logistic regression estimates: predictors of medium/high MedDiet adherence.

Variable	B	SE	Wald	p	OR (Exp(B))	95% CI
Saltiness level of foods	−1.344	0.333	16.253	<0.01**	0.261	[0.136, 0.501]
Sweetness level of foods	−0.713	0.318	5.044	0.025*	0.49	[0.263, 0.913]
Hypertension	0.865	0.492	3.098	0.078	2.376	[0.906, 6.226]
“I would follow the octagons, but nobody guides me”	0.254	0.129	3.907	0.048*	1.289	[1.002, 1.659]

Notes: Outcome: medium/high adherence (KIDMED 4–12 = 1). p-values from Wald test. SE = standard error. OR < 1 indicates lower odds of medium/high adherence as saltiness/sweetness intensity increases. Model fit: χ^2^(4) = 27.351, p < 00.001; Nagelkerke R^2^ = 0.173; overall accuracy = 63.0%. *p < 0.05; **p < 0.01.

## 4 Discussion

This study contributes original evidence by examining, in adults after bariatric surgery, the association between perceptions of front-of-pack (FOP) nutrition labeling and adherence to the MedDiet—an understudied population. Our findings indicate that medium/high MedDiet adherence is linked to greater agreement that the octagonal warning influences product choice; that stronger preferences for salty and sweet foods are inversely associated with the odds of medium/high adherence; and that a greater willingness to follow FOP warnings—even in the absence of formal guidance—relates to higher adherence. We also observed sex differences in selected KIDMED items, whereas cardiometabolic comorbidities (e.g., hypertension, dyslipidemia) did not show consistent associations after adjustment. Taken together, these results suggest that, beyond clinical status, the interplay between sensory preferences and the capacity to use simple point-of-purchase cues may shape dietary behavior during the postoperative period. They suggest potential areas for tailored educational interventions that combine brief education on FOP labels with sensory retraining (e.g., progressive reduction of salt and added sugars) to consolidate MedDiet -consistent patterns throughout follow-up. While the cross-sectional design and self-reported measures limit causal inference, the internal coherence of the effects and the use of previously established instruments, together with a preliminary internal psychometric assessment of the KIDMED index in this sample, adds interpretive value to these findings.

Because this study focused exclusively on the postoperative phase, our findings indicate that—despite the adoption of seemingly healthier eating practices—metabolic problems and excess weight remain common among most surgically treated patients. Recent literature concurs that many patients do modify eating behaviors after bariatric surgery, yet such changes are often partial or insufficiently sustained over time [31]. Furthermore, overall dietary quality, beyond isolated “nutrients of concern,” is decisive for maintaining weight improvements and remission of metabolic disturbances [32]. Even when positive changes occur, the body engages compensatory mechanisms—e.g., increased ghrelin or reduced energy expenditure—that complicate long-term weight maintenance [33], [34]. This helps explain why altering a subset of habits does not ensure durable outcomes and underscores the need to strengthen interdisciplinary follow-up, particularly nutrition education, clinical monitoring, and psychological support, to consolidate metabolic benefits and prevent relapse [35].

A salient finding is that although bariatric patients report high acceptance of front-of-pack (FOP) warning-label design, many cannot interpret it correctly and lack guidance to apply it in food choices. This perception–use gap has been documented, indicating that the effectiveness of warning systems is constrained by low nutrition literacy [28,36]. While the octagonal stop sign fulfills its initial deterrent function by capturing attention and flagging products high in sugar, sodium, or saturated fat, its real-world utility depends on users’ ability to understand the underlying nutrient profile. The literature therefore recommends pairing FOP labels with educational strategies that place the warning within a broader dietary context. Accordingly, FOP labels may function as a useful alert, but without practical education their impact on eating behavior remains limited [37].

In bivariate analyses, medium/high MedDiet adherence was more frequent among participants with normal BMI status than among those with obesity, whereas stronger preferences for salty and sweet foods were associated with lower adherence. However, BMI status was not retained in the final multivariable model, suggesting that its relationship with MedDiet adherence should be interpreted cautiously in this sample. This pattern is partly consistent with studies linking higher MedDiet adherence to more favorable postoperative outcomes, including weight-related indicators [38]. Conversely, other reports describe low adherence to healthy patterns at one year [39], which may depend on the nutrition counseling provided and individual motivation [40]. In addition, patients with greater preoperative obesity have been shown to adopt more favorable eating behaviors [41], possibly as an adaptive response to their prior status. Physiological and behavioral changes after surgery also reshape taste responsiveness—reduced preference for sweet foods [42] and increased sensitivity to saltiness [43]—thereby contributing to dietary behavior change [44]. Taken together with motivational factors, these mechanisms may facilitate sustained adherence to MedDiet-consistent patterns [45].

Our results also indicate that sex and perceived weight status influence diet quality. Specifically, women exhibited a mixed pattern: higher intake of legumes and staple cereals (pasta/rice) alongside greater breakfast pastry consumption, an ultra-processed item. This duality may reflect a combination of sociocultural factors, personal preferences, and entrenched habits that persist after bariatric surgery. Consistent with reports from other populations, women tend to consume more healthful foods (vegetables, fruits, legumes, whole grains) yet also report more sugary snacks and sugar-sweetened beverages, yielding a not fully balanced pattern [46]. The literature further describes oscillations between health-oriented choices (self-control, body-image concerns) and emotion- or craving-driven intake of ultra-processed foods [47]. In contrast, no significant differences were observed by NCD status, suggesting that subjective weight appraisal may shape day-to-day choices more than the mere presence of a clinical diagnosis.

No significant associations were detected between sex, perceived excess weight, or NCD status and overall perceptions of front-of-pack (FOP) labeling. However, individuals not perceiving themselves as overweight scored significantly higher on the item assessing whether the octagonal warning influences product choice, which may reflect a greater reported influence of labeling on purchasing decisions in this subgroup. This pattern partly diverges from general-population reports describing broader positive effects of FOP on healthier choices [48].

Variables such as agreement with label design and the influence of price/promotions yielded odds ratios above one but did not reach statistical significance, precluding firm inferences. Likewise, nutrition-knowledge indicators (e.g., recommended salt/sugar levels) were not associated with adherence to the MedDiet. One possible explanation is that, in postoperative bariatric care, dietary behavior may be shaped by multiple factors beyond nutrition knowledge alone, including ongoing multidisciplinary follow-up, behavioral adaptation, and individual adherence trajectories [45]. Statistical power may also have been constrained by sample size.

Belief that the octagonal FOP warning influences product choice was positively associated with MedDiet adherence. This pattern may indicate that individuals who report greater relevance of FOP messages also report healthier eating patterns. In this context, FOP labels may be interpreted as a point-of-purchase cue associated with choices more consistent with a balanced dietary pattern. The finding is consistent with evidence showing that FOP improves understanding and reduces purchase intentions for unhealthy products [49]. Nevertheless, effectiveness is conditioned by modulating factors—notably nutrition education and cultural practices [50]; therefore, FOP should be embedded within broader intervention packages (e.g., nutrition counseling and behaviorally informed communication).

Binary logistic regression identified variables significantly associated with MedDiet adherence. Notably, frequent intake of salty and sweet foods was negatively associated with adherence, consistent with evidence that ultra-processed foods—high in sodium, sugars, and fats—displace healthy patterns and increase the risk of noncommunicable diseases (Monteiro et al., 2019; Khandpur et al., 2018). This finding underscores the contrast between an MedDiet centered on fresh, minimally processed, nutrient-dense foods and westernized, ultra-processed eating patterns. By contrast, the lack of a significant association between hypertension status and adherence suggests that the mere presence of a chronic condition does not necessarily translate into behavioral change; structural barriers, limited nutrition education, and practical challenges to sustaining dietary modifications are plausible explanations [51].

In contrast, a greater willingness to follow front-of-pack (FOP) octagonal warnings was positively associated with MedDiet adherence, a finding that may be related to individual motivational factors. This finding aligns with evidence supporting the effectiveness of FOP labeling in promoting healthier choices, particularly when combined with nutrition education and well-designed communication strategies [52,53]. Although the multivariable model exhibited moderate explanatory power, its significance indicates that perceptual and behavioral variables—such as label use and consumption habits— could be considered in the design of future educational strategies aimed at improving diet quality. Future research should incorporate contextual factors—including socioeconomic environment, availability of healthy foods, and nutrition literacy—to more fully elucidate determinants of adherence to healthy dietary patterns [54].

## 5 Implications

These findings extend knowledge on front-of-pack labeling in bariatric patients and reveal a perception–comprehension–use gap, suggesting a possible framework in which label perception, comprehension, and use may be related to dietary adherence, moderated by nutrition literacy, cultural practices, and postoperative stage. Practically and for policy, pre- and post-surgical structured FOP-literacy modules could be considered, “label-reading competence” adopted as a quality metric in bariatric follow-up, counseling tailored to postoperative cognitive load, and multicomponent interventions (brief counseling, digital nudges, healthy swap lists) delivered by multidisciplinary teams, alongside reinforced public campaigns and culturally adapted messaging. Future research should prioritize longitudinal and trial designs to test causal effects of labeling on adherence, examine mediators and moderators (literacy, procedure type, socioeconomic context), and assess the cost-effectiveness of these strategies.

## 6 Limitations

This study has several limitations. First, the cross-sectional design precludes causal inference and does not capture trajectories of change; pre-operative measures were unavailable, preventing before–after comparisons. Second, key variables on perceptions and intake relied on self-report, introducing recall and social-desirability bias; the FOP-perception instrument showed moderate internal consistency, raising the possibility of measurement error. An additional limitation is that the KIDMED index was originally developed for children and adolescents rather than adults after bariatric surgery. Although a preliminary internal psychometric evaluation was conducted in this sample, its internal consistency was only modest and its internal structure was not clearly unidimensional. Therefore, its use in this study should be considered exploratory. Third, a non-probability sample from a single center in Lima limits external validity to other clinical settings, regions, and bariatric procedures. Fourth, although the type of bariatric procedure was described, it was not incorporated into the multivariable models, and several potential confounders were not included, particularly social support, nutrition literacy, motivation, and other postoperative clinical factors. Fifth, objective markers of dietary behavior (e.g., real purchase data, dietary biomarkers) were not collected. Future research should use longitudinal designs with pre/post assessments, multicenter probability sampling, objective behavioral measures, and incorporate psychosocial/contextual factors, as well as trials testing education packages integrated with front-of-pack labeling.

## 7 Conclusion

Among bariatric surgery patients, MedDiet adherence was chiefly associated with behavioral and perceptual factors—a lower preference for highly salty/sweet foods and a greater willingness to follow front-of-pack warnings—whereas sociodemographic variables showed weak relationships, likely attenuated by postoperative physiological and behavioral shifts. These findings suggest that FOP labeling may have practical value as a point-of-decision educational cue and highlight the relevance of strengthening label literacy into postoperative care. Sex differences and perceived overweight emerged as potential modulators of food choices; addressing these dimensions may enhance tailored postoperative interventions aimed at sustaining a Mediterranean-style dietary pattern.

## 8 Clinical key messages

• Front-of-pack warning labels may serve as a practical decision aid for bariatric patients, supporting day-to-day selection of healthier foods consistent with MedDiet guidelines.
• Postoperative taste preferences—particularly reduced preference for salty and sweet foods—are strongly associated with healthier dietary patterns and may be leveraged during nutritional counseling.
• Integrating brief, structured label-literacy education into routine bariatric follow-up could enhance long-term diet quality and strengthen postoperative adherence to Mediterranean-style eating.

## Ethics approval and consent to participate

The study was approved by the Research Ethics Committee of the Faculty of Health Sciences at Universidad Peruana Unión (protocol ID 2025-CE-EPG-00046). All procedures followed the ethical principles of the Declaration of Helsinki and applicable national regulations. Written informed consent was obtained from all participants prior to data collection.

## Availability of data and material

All relevant data are presented in the main paper.

## Authors' contributions

SMLM and LPPR contributed to the study conceptualization, design, and data collection. WCMG contributed to the interpretation of data and drafting of the manuscript. YECM provided methodological supervision, critically reviewed the manuscript for intellectual content, and approved the final version for submission. All authors read and approved the final manuscript.

## Declaration of artificial intelligence (AI) and AI-assisted technologies utilized in the writing process

None.

## Funding

This research did not receive external funding. The publication fees for this article will be covered by the Universidad Peruana Unión.

## Declaration of competing interest

The authors declare that they have no known competing financial interests or personal relationships that could have appeared to influence the work reported in this paper.
