# Supplementary material for: Association of front-of-package warning label perceptions with Mediterranean diet adherence after bariatric surgery: FOP perceptions and MedDiet adherence post-bariatric surgery
Source: Obes Pillars. 2026 Apr 14;18:100266. doi: 10.1016/j.obpill.2026.100266 (PMC13101637; doi:10.1016/j.obpill.2026.100266)
Supplement: Multimedia component 1 [file mmc1.docx]

**Supplementary Table S1. KIDMED index items used in this study**

Supplementary Table S1. Structure of the KIDMED index used to assess Mediterranean-diet-related behaviors in adults after bariatric surgery

| **Code** | **Brief item label** | **Item orientation** | **Scoring rule** | **Response options** |
| --- | --- | --- | --- | --- |
| P22 | Fruit or fruit juice every day | Positive | Yes = +1; No = 0 | Yes / No |
| P23 | A second fruit every day | Positive | Yes = +1; No = 0 | Yes / No |
| P24 | Fresh or cooked vegetables once a day | Positive | Yes = +1; No = 0 | Yes / No |
| P25 | Fresh or cooked vegetables more than once a day | Positive | Yes = +1; No = 0 | Yes / No |
| P26 | Fish regularly (at least 2–3 times/week) | Positive | Yes = +1; No = 0 | Yes / No |
| P27 | Fast-food restaurant more than once/week | Negative | Yes = −1; No = 0 | Yes / No |
| P28 | Legumes more than once/week | Positive | Yes = +1; No = 0 | Yes / No |
| P29 | Pasta or rice almost daily | Positive | Yes = +1; No = 0 | Yes / No |
| P30 | Cereals or grains for breakfast | Positive | Yes = +1; No = 0 | Yes / No |
| P31 | Nuts regularly (at least 2–3 times/week) | Positive | Yes = +1; No = 0 | Yes / No |
| P32 | Olive oil used at home | Positive | Yes = +1; No = 0 | Yes / No |
| P33 | Skips breakfast | Negative | Yes = −1; No = 0 | Yes / No |
| P34 | Dairy product for breakfast | Positive | Yes = +1; No = 0 | Yes / No |
| P35 | Pastries/cookies for breakfast | Negative | Yes = −1; No = 0 | Yes / No |
| P36 | Two yogurts and/or some cheese daily | Positive | Yes = +1; No = 0 | Yes / No |
| P37 | Sweets/candy several times per day | Negative | Yes = −1; No = 0 | Yes / No |

**Note:** The KIDMED index includes 16 dichotomous items. Positive items are scored +1 and negative items are scored −1; negative responses are scored 0. Total scores range from −4 to 12. In this study, KIDMED scores were dichotomized as low adherence and medium/high adherence, according to the analytical strategy described in the Methods section. Because the KIDMED index was originally developed for children and adolescents, it was used here as a proxy measure of Mediterranean-diet-aligned behaviors in adults after bariatric surgery.

**Supplementary Table S2. Front-of-package warning-label perception questionnaire used in this study**

Supplementary Table S2. Front-of-package warning-label perception questionnaire and additional dietary/clinical items used in this study

| **Code** | **Item / question** | **Domain** | **Response options** |
| --- | --- | --- | --- |
| P11 | The octagonal warning label on the package influences my product choice | Perceived influence on choice | Strongly disagree / Disagree / Neither agree nor disagree / Agree / Strongly agree |
| P12 | Prices and promotions influence my purchase decisions | Competing purchase influences | Strongly disagree / Disagree / Neither agree nor disagree / Agree / Strongly agree |
| P13 | I would follow the octagons, but I do not have time to read them | Barrier: time | Strongly disagree / Disagree / Neither agree nor disagree / Agree / Strongly agree |
| P14 | I would follow the octagons, but I do not know how to interpret them | Barrier: interpretation | Strongly disagree / Disagree / Neither agree nor disagree / Agree / Strongly agree |
| P15 | I would follow the octagons, but I receive no guidance on how to use them | Barrier: guidance | Strongly disagree / Disagree / Neither agree nor disagree / Agree / Strongly agree |
| P16 | I agree with the implementation of octagons in Peru | Policy support | Strongly disagree / Disagree / Neither agree nor disagree / Agree / Strongly agree |
| P17 | I am satisfied with the label design/model | Label acceptability | Strongly disagree / Disagree / Neither agree nor disagree / Agree / Strongly agree |
| P18 | I know the recommended intake level for salt | Self-perceived nutrition knowledge | Strongly disagree / Disagree / Neither agree nor disagree / Agree / Strongly agree |
| P19 | I know the recommended intake level for sugar | Self-perceived nutrition knowledge | Strongly disagree / Disagree / Neither agree nor disagree / Agree / Strongly agree |
| P20 | I know the recommended intake level for saturated fat | Self-perceived nutrition knowledge | Strongly disagree / Disagree / Neither agree nor disagree / Agree / Strongly agree |
| P5 | Saltiness preference | Dietary preference | Little/not salty / Very/regularly salty |
| P6 | Sweetness preference | Dietary preference | Little/not sweet / Very/regularly sweet |
| P7 | Hypertension | Clinical history | No / Yes |
| P8 | Type 2 diabetes | Clinical history | No / Yes |
| P9 | Dyslipidemia | Clinical history | No / Yes |

**Note:** The front-of-package warning-label perception questionnaire included 10 Likert-type items assessing perceived usefulness, barriers, acceptance, and self-perceived nutrition knowledge regarding Peruvian octagonal warning labels, plus additional items on saltiness preference, sweetness preference, and noncommunicable disease history. Higher scores on Likert items indicate stronger agreement.
